# Unilateral renal mucormycosis in a patient presenting with pyelonephritis and acute kidney failure: A case report

**DOI:** 10.1002/ccr3.8950

**Published:** 2024-05-26

**Authors:** Bahar Darouei, Mohammad Mehdi Zare, Hedie Torkashvan, Abbas Ali Torfeh Esfahani

**Affiliations:** ^1^ School of Medicine Isfahan University of Medical Sciences Isfahan Iran; ^2^ Department of Infectious Diseases and Tropical Medicine, School of Medicine Isfahan University of Medical Sciences Isfahan Iran

**Keywords:** acute kidney injury, case report, cystinuria, mucormycosis, nephrolithiasis

## Abstract

**Key Clinical Message:**

Unilateral renal mucormycosis is a rare infection that should be suspected in patients with recurrent renal infections presenting nonspecific clinical features that do not respond to conventional therapies, especially in impaired immune systems due to related risk factors. Moreover, histopathological examinations should be performed to confirm the diagnosis. For treatment, the preference is that the patient is hospitalized, and surgical intervention and rapid administration of intravenous antifungals for 2–3 weeks are the treatment choices. After discharge, the patient should be followed up with periodic blood urea nitrogen and creatinine levels and, if needed, an imaging modality such as a CT scan or sonography.

**Abstract:**

Renal mucormycosis (RM) is a rare form of mucormycosis infection and is more often in immunocompromised patients with risk factors. Unilateral renal involvement is infrequent in patients and is available as case reports. This condition usually presents with renal colic, fever and chills, and oliguria and has a high mortality rate. Herein, we report a case of unilateral renal mucormycosis presenting with pyelonephritis and acute kidney injury in a 32‐year‐old patient. The patient had numerous urological procedures in previous years due to nephrolithiasis state, which put him in an immunocompromised state. The histopathological examination of the pylocalyceal system revealed a collection of broad non‐septated fungal hyphae branching at 90° accompanied by numerous neutrophils and necrotic tissue in favor of mucormycosis. He was successfully treated with 5 mg/kg/day Liposomal Amphotericin B for 3 weeks, discharged with good general condition, and remained asymptomatic for 3 months after discharge. The diagnosis of RM relies on solid clinical suspicion, which can be authenticated by histopathological examination, and the combination of antifungal therapy and surgical intervention can result in a good outcome.

## 1 INTRODUCTION

Mucormycosis is a rare fungus infection with high mortality caused by *Mucorales*.^1^, ^2^ Mucormycosis mainly causes infection in immunocompromised patients with predisposing factors, including tissue transplantation, diabetes, hematologic malignancies, immunosuppressive drugs, surgeries, trauma, and Covid‐19.^3^, ^4^ The exact mechanism of RM remains unclear; however, retrograde spread from lower urinary tract infections and hematogenous dissemination to the kidneys is suggested.^5^ Disseminated forms of mucormycosis leading to renal involvement have been observed in up to 20% of cases; however, unilateral renal mucormycosis (RM) is infrequent and available as case reports.^6^, ^7^ Herein, we present a case of unilateral RM after frequent urologic procedures due to recurrent nephrolithiasis status caused by cystinuria in an adult man. In this case, we aim to emphasize mucormycosis's multifaceted nature and consider fungal infections, even atypical clinical presentations, especially in individuals with underlying predisposing conditions.

## 2 CASE HISTORY

A 32‐year‐old man previously diagnosed with nephrolithiasis and chronic kidney disease (CKD) presented to our hospital complaining of fever and chills, renal colic, and urinary obstruction. He was an immunocompetent healthy man with no past medical history until 5 years ago when, after an episode of renal colic, he was diagnosed with multiple bilateral cystine renal stones and staghorn stones. At the time, nephrolithiasis was found to be so severe that it made his right kidney atrophic.

**FIGURE 1:**
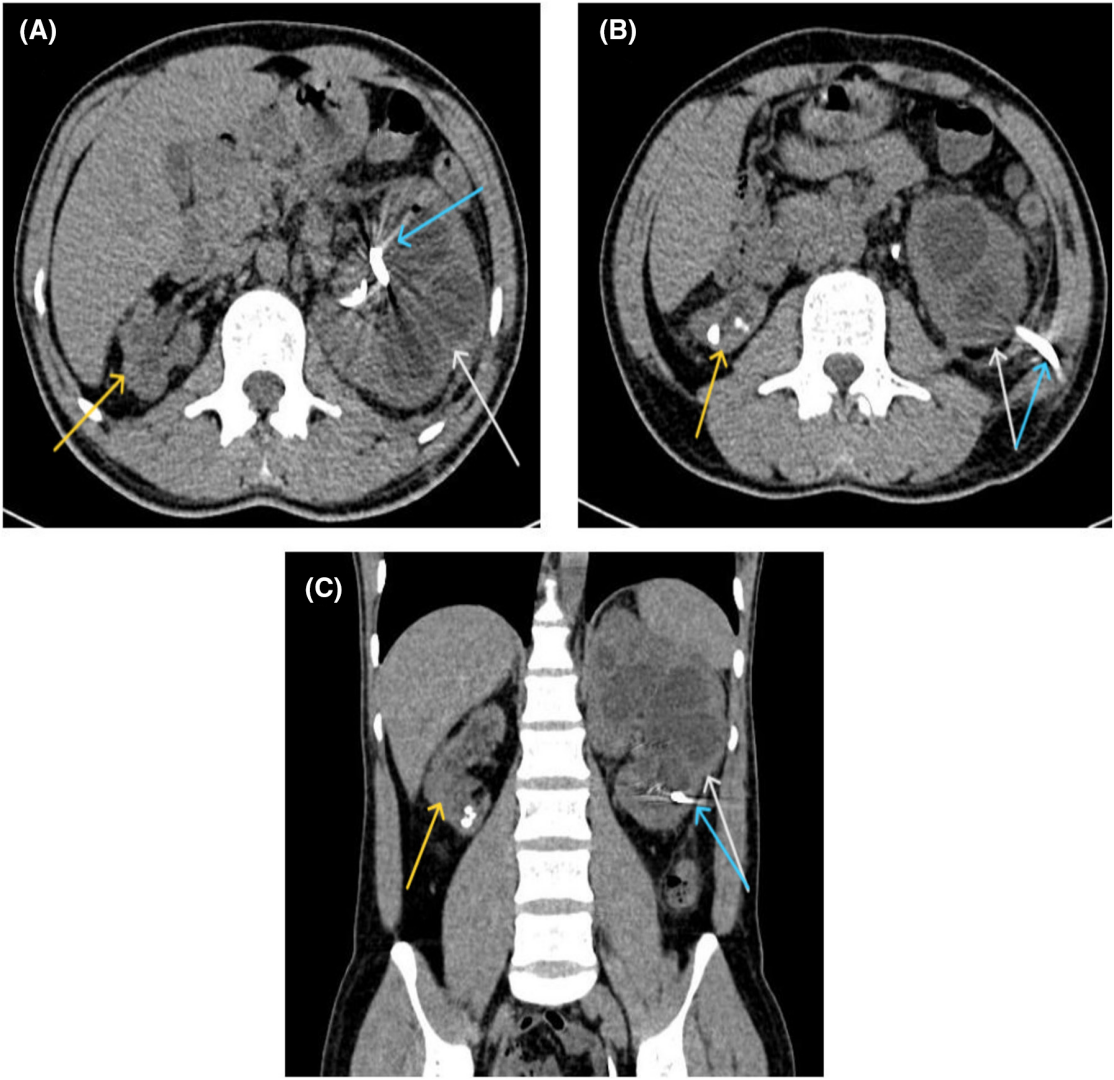
Axial (A, B) and coronal (C) views of the multi‐detector CT scan show enlargement of the left kidney mostly compatible with pyelonephritis (white arrow), the atrophic appearance of the right kidney with renal stones (yellow arrow), and a left‐sided nephrostomy and left double J catheter (blue arrow) at the time the patient presented with acute kidney injury.

During these 5 years, he underwent several urologic procedures such as percutaneous nephrolithotomy (PCNL), transurethral lithotripsy (TUL), and extracorporeal shock wave lithotripsy (ESWL) due to recurrent renal stones (seven times PCNLs, two times ESWL, and one‐time TUL). In the recent year, fever and chills, infection at the procedure site, and creatinine rise were also added to the patient's previous symptoms and led to his long‐term hospitalization (3 times in 6 months). During each hospitalization, the patient was treated with intravenous antibiotics and antifungals along with double J and nephrostomy replacement, which was ineffective and caused him to be hospitalized for a short while after discharge. In his last hospitalization at Day −35, in addition to the previous actions, PCNL was performed to drain pus and take samples from the renal‐pelvic system. Acute inflammation was reported, with no evidence of malignancy observed. At this time at Day −32, abdominopelvic Multi‐Detector Computed Tomography (MDCT) was obtained, which revealed several bilateral kidney stones, atrophy of the right kidney (length of 79 mm), and severe hydronephrosis of the left kidney (length of 143 mm) suggestive of pyelonephritis (Figure 1). Despite numerous hospitalizations and going under treatments—thoroughly explained above—the patient developed CKD with a creatinine base of 3 mg/dL.

In our center, the patient presented again with fever and chills, urinary obstruction, and renal colic. He mentioned no current use of medication and a family history of nephrolithiasis in his sister and aunt. On physical examination at Day 0, the patient was febrile (*T* = 38.4°C), with a heart rate of 87/min, blood pressure of 120/85 mmHg, oxygen saturation of 95%, and left flank tenderness. Laboratory findings on admission revealed hemoglobin of 11.3 g/dL, hematocrit of 33.6%, white blood cell count (WBC) of 13,900 cell/mm^3^, blood urea nitrogen of 29 mg/dL, creatinine of 4.9 mg/dL, and C‐reactive protein of 93 mg/dL. Urinalysis at Day +1 showed protein 2+, blood +, leukocyte esterase positive, RBC 10–12, and WBC 28–30, but the urine culture was sterile. Immunologic tests were within normal ranges, including C3, C4, anti‐dsDNA, antinuclear antibody (ANA), C‐ANCA, P‐ANCA, and anti‐glomerular basement membrane (anti‐GBM) antibody. Chest radiograph revealed no pathologic finding.

He was started with intravenous antibiotics and underwent another PCNL at Day +3, and during the procedure, a fungal bezoar was observed in the left pyelocalyceal system.

## 3 DIFFERENTIAL DIAGNOSIS, INVESTIGATIONS AND TREATMENT

**FIGURE 2:**
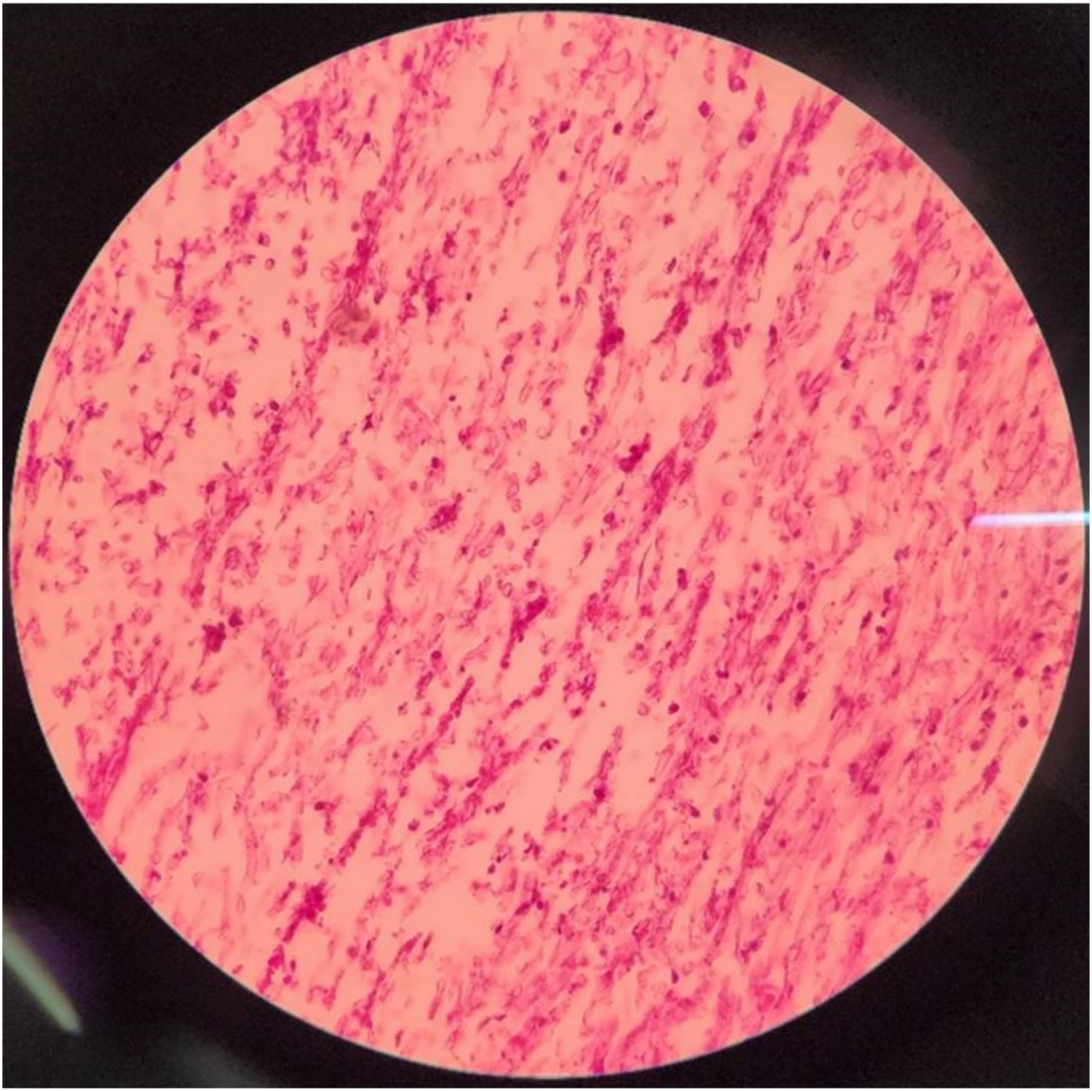
Microscopic specimen from the fungal bezoar in the left pyelocalyceal system was stained by H&E and PAS stains, demonstrating an extensive collection of broad non‐septated fungal hyphae branching at 90° accompanied by numerous neutrophils and necrotic tissue in favor of Mucormycosis (40×).

Differential diagnoses of this renal bezoar formation included tuberculosis, fungal infection, and renal cell carcinoma. Biopsy was taken, and double J and nephrostomy were implanted in the left kidney due to post‐renal azotemia. Histopathological examination at Day +10 revealed blood clots and necrotic tissue with extensive broad ribbon‐like and non‐septated fungal hyphae branching at 90°, accompanied by numerous neutrophils favoring mucormycosis (Figure 2). The patient was started on Liposomal Amphotericin B (LAMB) with the dosage of 5 mg/kg/day for 21 days at Day +11.

## 4 OUTCOME AND FOLLOW‐UP

On Day +33, the patient's serum creatinine was reduced to 3.5 mg/dL, the double J and nephrostomy were removed, and he was discharged in good general condition. Three months after the discharge, the patient is still asymptomatic with stable serum creatinine (2.5 mg/dL). The follow‐up urinalysis showed protein 2+, blood trace, leukocyte esterase negative, nitrite negative, RBC 1–2, and WBC 0–1.

## 5 DISCUSSION

RM is an uncommon kind of mucormycosis that invades the blood vessels and causes vascular thrombosis and renal ischemic necrosis, which is reported in a few cases.^8^ RM is more prevalent in Asia, especially India, with preponderance in males with a mean age of 33.^5^ The precise underlying mechanism of RM is not well understood; however, ascending movement of RM from the lower urinary tract to the kidney and spreading throughout the bloodstream are possible mechanisms.^9^ The hallmark clinical features of RM encompass fever, flank pain (either unilateral or bilateral), hematuria, anuria, oliguria, and pyuria.^5^, ^8^ Due to nonspecific clinical features, most of the patients with RM can be misdiagnosed as acute pyelonephritis and be treated by antibiotics.^6^, ^8^ However, treatment failure can provide a clue for further investigation and raise the diagnosis of fungal infection.^6^, ^8^ After clinical suspicion, the most practical modalities are CT and ultrasonography, which often show findings favoring enlarged kidneys, hydronephrosis, and pyelonephritis.^5^ To confirm the diagnosis, the histopathologic examination plays a crucial role in the diagnosis of RM, demonstrating Ribbon‐like and aseptate hyphae of Mucor, with surrounding tissue necrosis.^5^

Different RM management pathways can be applied in solitary or in combination. The mainstay approach to RM is a combination of surgery and antifungal therapy; however, either of them can be used alone. Amphotericin B, Posaconazole, and isavuconazole are well‐known drugs that have shown efficacy in treating mucormycosis.^10^ Among amphotericin B formation, LAMB has shown better tolerability and fewer adverse effects nephrotoxicity than deoxycholate amphotericin B.^11^ The optimal dosage for amphotericin B in mucormycosis infection is challenging considering drug's side effects and nephrotoxicity, so the recommended dosage for LAMB is 5 mg/kg/day in the absence of central nervous system.^11^ Nevertheless, administering LAMB in acute kidney injury necessitates high‐quality supporting care and close monitoring to maintain or restore the function of the kidney's distal tubular cells.^12^ However, patients do not respond well to the anti‐fungals in the RM case reports requiring nephrectomy or percutaneous nephrostomy drainage in over 50% of cases.^13^

RM has a poor prognosis, with a high mortality rate between 44% and 85%.^5^ The outcome relies on several factors, such as the severity and extent of the infection, the type of Mucorales involved, the promptness and appropriateness of diagnosis and treatment, and the existence and management of underlying conditions.^14^ The major reasons for death in RM are septic shock, renal failure, and the spread of the infection to other organs. Therefore, it is vital to diagnose and treat RM as soon as possible to reduce the risk of complications and death.^4^, ^5^ Risk factors for RM infection are mainly due to immunocompromised status, such as organ transplantation recipients, patients with HIV or uncontrolled diabetes mellitus, malignancy, and intravenous drug abusers.^15^ The noteworthy point in our patient is the absence of one of these risk factors. On the other hand, this patient was diagnosed with cystinuria along with multiple bilateral kidney stones for the first time at the age of 27.

*Cystinuria* is a hereditary disorder that causes severe nephrolithiasis in children, although diagnosis in adulthood is not uncommon.^16^ Cystine stones are often large, and recurrences happen frequently.^17^ Hence, these patients might require multiple stone removal procedures throughout their lives, which causes an elevated risk of developing CKD and renal failure, not only compared to the general population but also in comparison to other individuals who form stones.^17^, ^18^

The case described above is unique in the sense that this patient was previously immunocompetent with no significant risk factor for RM. However, due to cystinuria, he started to develop multiple bilateral kidney stones and, therefore, kidney insufficiency. He was exposed to this rare disease due to renal impairment, facilitating fungus invasion. Also, his constant exposure to invasive urologic procedures could enhance the risk of infection as well and put him in an immunocompromised state. Our case demonstrates the complexities of diagnosis RM due to nonspecific symptoms and signs such as fever, chills, renal colic, and urinary obstruction that might be explained in the background of his nephrolithiasis, pyelonephritis, or renal failure. Also, this case points to the importance of early diagnosis of cystinuria and the need to utilize medical treatments as much as possible to minimize invasive procedures which can which can weaken the body's immune system and facilitate opportunistic infections.

## 6 CONCLUSION

Our case describes unilateral RM in an immunocompromised individual with no known underlying health conditions which was susceptible to this rare infection due to numerous urological procedures. The diagnosis of RM depends on strong clinical suspicion, which can be confirmed by histopathological examinations. Surgical intervention and rapid treatment with intravenous antifungals such as liposomal amphotericin B can result in good outcomes in these patients.

## AUTHOR CONTRIBUTIONS

**Bahar Darouei:** Conceptualization; data curation; project administration; writing – original draft. **Mohammad Mehdi Zare:** Data curation; writing – original draft. **Hedie Torkashvan:** Data curation; writing – original draft. **Abbas Ali Torfeh Esfahani:** Conceptualization; project administration; supervision; writing – review and editing.

## FUNDING INFORMATION

None.

## CONFLICT OF INTEREST STATEMENT

All the authors have declared no competing interest.

## ETHICS STATEMENT

Not applicable.

## CONSENT

Written informed consent was obtained from the patient to publish this report in accordance with the journal's patient consent policy.

## Data Availability

Data sharing is not applicable to this article as no new data were created or analyzed in this study.
